# Segmental outflow dynamics in the trabecular meshwork of living mice

**DOI:** 10.1016/j.exer.2022.109285

**Published:** 2022-10-21

**Authors:** Ester Reina-Torres, Tiffany M.G. Baptiste, Darryl R. Overby

**Affiliations:** Department of Bioengineering, Imperial College London, London, United Kingdom

**Keywords:** Trabecular meshwork, Segmental outflow, Mouse models, Aqueous humour outflow

## Abstract

Aqueous humour does not drain uniformly through the trabecular meshwork (TM), but rather follows non-uniform or “segmental” routes. In this study, we examined whether segmental outflow patterns in the TM change over time in living mice and whether such changes are affected by age. Segmental outflow patterns were labelled by constant-pressure infusion of fluorescent tracer microparticles into the anterior chamber of anesthetised C57BL/6J mice at 3 or 8 months of age. Two different tracer colours were infused at separate time points with an interval of Δt = 0, 2, 7 or 14 days. In a separate experiment, one tracer was infused *in vivo* while the second tracer was infused *ex vivo* after 2 days. The spatial relationship between the two tracer patterns was analysed using the Pearson’s correlation coefficient, *r*. In 3-month-old mice, there was a time-dependent decay in *r*, which was near unity at Δt = 0 and near zero at Δt = 14 days. In 8-month-old mice, *r* remained elevated for 14 days. Segmental outflow patterns measured in young mice *ex vivo* were not significantly different from those measured *in vivo* after accounting for the expected changes over 2 days. Therefore, segmental outflow patterns are not static in the TM but redistribute over time, achieving near complete loss of correlation by 2 weeks in young healthy mice. There is an age-related decline in the rate at which segmental outflow patterns redistribute in the TM. Further research is needed to understand the dynamic factors controlling segmental outflow.

## 1. Introduction

Aqueous humour outflow through the trabecular meshwork (TM) is the primary determinant of intraocular pressure (IOP), and increased aqueous humour outflow resistance is the root cause of IOP elevation in glaucoma (Grant, 1951). However, the hydrodynamic details of how aqueous humour flows through the TM and the factors contributing to outflow resistance generation remain poorly understood.

It is now well recognised that aqueous humour does not drain uniformly through the TM, but rather follows non-uniform or “segmental” routes such that only a fraction of the TM is filtration active at any given instant (as summarised in prior reviews (Carreon et al., 2017; Overby et al., 2009; Swaminathan et al., 2014)). There is a long history of studies examining fluid drainage patterns through the TM in eyes from several different species, including monkey (Epstein and Rohen, 1991; Sabanay et al., 2000), human (Braakman et al., 2015; Cha et al., 2016; Chang et al., 2014; de Kater et al., 1989; Ethier and Chan, 2001; Hann et al., 2005; Hann and Fautsch, 2009; Keller et al., 2011; Swain et al., 2022; Yang et al., 2013), mouse (Carreon et al., 2017; Li et al., 2016; Swaminathan et al., 2013) and bovine (Battista et al., 2008; Lu et al., 2008; Zhu et al., 2010), using either endogenous pigment (Johnson, 1989) or following perfusion with exogenous flow tracers, such as cationic or anionic ferritin (de Kater et al., 1989; Epstein and Rohen, 1991; Ethier and Chan, 2001; Hann et al., 2005; Melamed et al., 1986), thorium dioxide (Thorotrast) (Inomata et al., 1972; Tripathi, 1971), colloidal gold (Overby et al., 2002; Sabanay et al., 2000), latex microspheres (Huggert, 1957; Johnson et al., 1990) or fluorescent tracer microparticles (Braakman et al., 2015; Carreon et al., 2017; Cha et al., 2016; Chang et al., 2014; Li et al., 2016; Swain et al., 2022; Swaminathan et al., 2013; Vranka and Acott, 2017; Vranka et al., 2020). Pigment and tracer particles likely accumulate in the TM due to filtration as the flow carrying these particulates passes through the outflow pathway. Regions of greater local accumulation of tracer (or pigment) are typically interpreted as regions of greater local filtration velocity, which presumably reflects variations in the local hydraulic conductivity of the TM or downstream tissues. The spatial variation in tracer labelling patterns within the TM thus provides a spatial representation of variations in local outflow conductivity at the time of tracer infusion. With rare exception (Vranka et al., 2020), most prior studies examined outflow patterns at individual time points or between two time points separated by a brief experimental stimulus (Chang et al., 2014; Zhu et al., 2010). It thus remains an open question whether the filtration-active regions of the TM are static and remain constant over time or whether these patterns redistribute dynamically in the living eye.

In this study, we investigated whether segmental outflow patterns vary over time in living mice, examining a time interval ranging from 2 days to 2 weeks. To label segmental outflow, we infused fluorescent tracer microparticles into the anterior chamber of C57BL/6J mice under anaesthesia. We used two different colours of tracer microparticles infused at two different time points, separated by a time interval, Δ*t*. We examined whether time-dependent changes in segmental outflow vary with age, and whether segmental outflow patterns measured *in vivo* match those measured in enucleated eyes *ex vivo*. While the term “segmental outflow” has been used to describe non-uniform flow over a range of different length scales and in different parts of the outflow pathway (e.g., distal vasculature (Saraswathy et al., 2016)), we restrict our attention to tracer labelling patterns in the TM, examining how these patterns vary over length scales spanning a few hundred micrometres to several millimetres around the full circumference of the TM in the mouse.

## 2. Materials and methods

### 2.1. Animals

Experiments on younger mice used male C57BL/6J mice purchased from a commercial supplier (Charles River Laboratories, UK) that were 10–13 weeks of age at the time of the first tracer infusion (n = 19). Experiments on older mice used male and female retired breeders from an NOS3-GFP reporter line (van Haperen et al., 2003) that were 33–35 weeks of age at the time of the first tracer infusion (n = 3). These transgenic mice have an inserted GFP gene under control of the human NOS3 promoter, which drives GFP expression in all endothelial cells, but otherwise have normal expression of murine endothelial nitric oxide synthase (van Haperen et al., 2003). Mice had access to food and water *ad libitum* and were housed in individually ventilated cages (max. 5 mice per cage) with a 12-h light/dark cycle. Mice were treated in accordance with the U. K. Animals (Scientific Procedures) Act, 1986, under the authority of a U. K. Home Office Project License. Mice were allowed to acclimate to the housing environment for at least 1 week prior to any regulated procedures.

### 2.2. Fluorescent tracer microparticles

Fluorescent tracer microparticles were 200 nm diameter carboxylate-modified polystyrene microspheres (FluoSpheres^™^, ThermoFisher Scientific, UK) that were either dark red (660/680 nm) or blue (365/415 nm). These wavelengths exhibit minimal spectral overlap with GFP or between the two tracer colours (Supplemental Fig. 1). Tracer stock solution was sonicated and diluted into sterile-filtered PBS containing divalent cations to a working concentration of 1 × 10^11^ particles/ml (~0.06% v/v) that was used for infusion.

### 2.3. In vivo tracer infusion

Tracers were delivered *in vivo* via intra-cameral infusion at constant pressure of 15 mmHg under isoflurane anaesthesia. Cannulation needles were custom-made from glass capillaries (outer diameter = 1 mm, inner diameter = 0.58 mm; World Precision Instruments, USA) that were pulled using a micropipette puller (P2000, Sutter instruments, US) and broken to have a tip outer diameter of approximately 100 μm. Mice were anaesthetised with 3% isoflurane (Isocare, Animalcare Ltd., UK) and 1 l/min oxygen while mounted in a stereotaxic head holder (Model 923-B, Kopf Instruments, US). The mouse was placed on a heated platform to maintain body temperature. Prior to infusion, the mouse was injected subcutaneously with 5 mg/kg enrofloxacin antimicrobial (Baytril; Bayer Healthcare, Germany). Topical dilation drops (phenylephrine hydrochloride, 2.5% w/v, and tropicamide, 1% w/v; Bausch & Lomb, UK) were then applied to both eyes to minimise risk of the needle tip contacting the iris.

The following procedure was carried out under a dissection microscope with the eye directed upwards. Prior to infusion, the cornea was punctured using a sterile glass micro needle to remove a small volume (≈2 μl) of aqueous humour from the anterior chamber by capillarity. During the puncture, the needle direction was parallel to the iris and slightly above the limbus. The puncture needle was then removed, and the infusion needle was carefully threaded through the same opening in the cornea using a micromanipulator (World Precision Instruments, USA). The infusion needle was prepared by backfilling with fluorescent tracer microparticles at the working concentration (see above), and the infusion needle was connected to a reservoir set to a height of 20.4 cm H_2_O (= 15 mmHg) above the eye. The infusion was allowed to continue for 10 min, and a drop of PBS was added to the cornea at the start of infusion to avoid dehydration. After 10 min, the needle was removed from the eye and a drop of topical antibiotic (1% w/w Fucithalmic, LEO Pharma, Denmark) was administered to the cornea. The head of the mouse was turned, and the contralateral eye was infused in the same manner. When both eyes were infused with tracer, anaesthesia was halted, and the mouse was removed from the head holder and allowed to recover under supervision in a recovery cage set to 33 °C for 15 min before being returned to its housing cage.

*In vivo* infusions were performed in 22 mice. For younger mice, the time interval, Δt, between the first and second tracer was set either to Δt = 0, corresponding to simultaneous infusion of both tracers mixed together, each at the same working concentration (n = 3 mice), or with the two tracers perfused separately with an interval of Δt = 2, 7 or 14 days (n = 3 mice each). For older mice, the time interval was set to Δt = 14 days (n = 3 mice). For each mouse, both eyes were assigned the same value of Δt, with the tracer infusions performed consecutively for each eye during the same anaesthesia period. Paired eyes from the same mouse were treated as independent samples; this is justified because the tracer labelling patterns in the TM are independent between paired eyes (Supplemental Data). The order of the tracer colour was randomised between mice. Forty-eight hours after infusion with the second tracer, mice were euthanised by cervical dislocation. Eyes were enucleated and immersion fixed in 4% paraformaldehyde (PFA, ThermoFisher Scientific, UK) at 4 °C for 5 h. Eyes were then transferred to PBS at 4 °C to await further processing. In one additional set of experiments in younger mice, both tracers were infused simultaneously (Δt = 0) into both eyes, but the tracers were allowed to remain in the TM for 2 weeks prior to euthanisation and enucleation (n = 3 mice). In a second additional set of experiments in younger mice, each eye was infused with the first tracer *in vivo*, followed by euthanisation and enucleation 48 h later for infusion with the second tracer *ex vivo* (n = 4 mice; see below).

Due to the orientation of the mouse during *in vivo* infusion, the infusion needle punctured the right cornea (OD) in the inferior quadrant and punctured the left cornea (OS) in the superior quadrant. As the needle puncture sites were visible by fluorescence imaging, presumably due to tracer microparticles remaining in the corneal stroma, these locations were used to identify the quadrant orientation in the anterior segments following flat mount imaging (see below).

### 2.4. Ex vivo tracer perfusion

These experiments were conducted to compare tracer patterns *ex vivo* versus those labelled *in vivo*. These experiments used eyes that were infused with the first tracer 48 h prior to euthanisation (n = 4 mice), as described above. Mice were culled by cervical dislocation and the eyes were carefully enucleated and placed in PBS at room temperature to await infusion, no longer than 30 min. The eye was submerged in a PBS bath at 35 °C, and the anterior chamber was cannulated simultaneously with two pulled glass needle cannulas having 100 μm bevelled tips that were held together by custom-made capillary holder. One cannula was connected to a syringe pump configured for withdrawal (PHD ULTRA; Harvard Apparatus, USA) (Supplemental Fig. 2). Upstream of the second cannula there was a microfluidic chip connected to a reservoir. The microfluidic chip contained two separate microfluidic channels, used to minimise the dead volume upstream of the eye. One microchannel contained sterile-filtered PBS with 5.5 mM glucose and divalent cations (referred to as “DBG”), while the second microchannel contained DBG containing fluorescent tracer microparticles. A valve located upstream of the microfluidic chip determined which microchannel received flow. This set up allowed for rapid exchange of anterior chamber contents at controlled IOP, as described previously (Reina-Torres et al., 2020).

The eyes were allowed to acclimate for 20 min at 8 mmHg while perfused with DBG. The anterior chamber was then exchanged with DBG containing fluorescent tracer microparticles at the same working concentration as used for *in vivo* infusions. The exchange was conducted at a flow rate of 5 μl/min and an IOP of 8 mmHg for 20 min, followed by a 10-min infusion with tracer at 8 mmHg to mimic the conditions of the *in vivo* tracer infusion (which assumes an episcleral vessel pressure of 7 mmHg *in vivo*). The anterior chamber was then exchanged with DBG alone to remove tracer for 20 min at 5 μl/min, followed by perfusion without tracer for an additional 20 min. All perfusions were done at a constant pressure with the reservoir set to a height of 8 mmHg above the eye. Exchanges were done with the reservoir set to a height of 8 mmHg + *R_q_* × 5 μl/min above the eye, where *R_q_* is the hydraulic resistance of the cannula, typically 0.01–0.05 mmHg/(μl/min), as measured using iPerfusion (Sherwood et al., 2016). After the perfusion, eyes were immersion fixed in 4% PFA overnight at 4 °C.

### 2.5. Preparing the anterior segment for imaging

Eyes were first washed three times with PBS for 15 min each. Under a dissection microscope, we cleaned the eye of any extra-ocular tissues and then hemisected the eye at the equator, discarding the posterior segment and lens. The anterior segment was then carefully cleared of any remaining choroid, leaving the iris intact, and the anterior segment was cut into a clover-leaf pattern for flat-mounting. The anterior segment was then placed into a drop of mounting media (Fluoroshield, F6182, Sigma Aldrich, UK) on a glass slide with the external ocular surface facing upwards. A second drop of mounting media was added on top of the eye, and a cover slip was placed on top of the specimen. The coverslip was sealed with transparent nail varnish and allowed to dry overnight at 4 °C before imaging.

### 2.6. Imaging

**Fig. 1.**
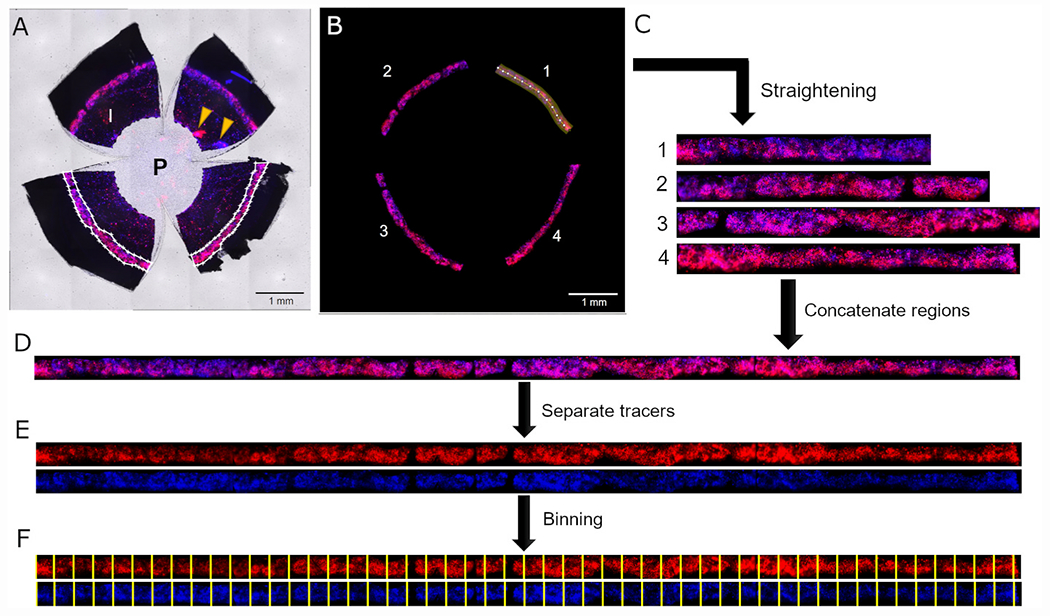
Workflow of the image processing and analysis using a representative eye that had tracers delivered two weeks apart. (A) Raw image acquired with a widefield microscope using tile imaging with red, blue and brightfield channels merged. The fluorescent tracers accumulate in the TM that appears as a disjointed fluorescent ring in the limbus. In white outlines, we have manually selected polygons that delineate each region of the TM (only two regions shown). I: iris; P: pupil; arrowheads indicate injection sites. (B) Masked image showing fluorescent signal from the TM only, removing all pixel data outside of the selected polygons. Each TM region was then straightened by projecting the fluorescent tracer image along piecewise line segments with a fixed width performed in ImageJ (the highlighted region shows one such piecewise line segment in the TM region labelled 1). This process was repeated for each of the four TM regions (C), which were then concatenated to obtain a straight continuous image of the fluorescent tracer patterns along the entire TM circumference (D). Identical processing steps were applied to both tracer images. The two tracer colours were then separated (E) and binned for co-localisation analysis, with the same binning process applied to each tracer colour (F).

Tracer patterns were imaged through the sclera using an inverted widefield fluorescence microscope with LED illumination (Zeiss Axio Observer, Germany). The microscope was configured to acquire sequential images of the two fluorescent tracer colours plus bright field at 10× magnification, using a tile-scan with a focus map to create a montage image that includes the entire anterior segment (Fig. 1A). We carefully selected the illumination power and exposure time to ensure that the tracer intensity was not saturated in the image.

### 2.7. Image analysis

Each tracer image was background corrected by subtracting the mean intensity value measured from three regions of interest (200 px x 200 px), placed at one location in the central cornea and two locations within the sclera. This was done using the interactive polygon tool in MATLAB (Mathworks; R2018a and R2021a).

In flat mount images, the TM appeared as 4 regions separated by manual cuts (Fig. 1A). We then applied digital tools to reassemble these regions to create one continuous rectangular image showing the full TM around its entire circumference. To do this, we first applied an interactive polygon tool in MATLAB to select a polygonal region of interest surrounding each of the four TM regions (white outlines in Fig. 1A). Any pixel within the polygons was defined to lie within the domain of the TM, while any pixel outside the polygons was defined to lie outside the TM domain. We then manually drew piecewise line segments along the central axis of each TM region (highlighted region in Fig. 1B) and projected the fluorescent tracer image onto this line segment using the “straighten” function in ImageJ (U. S. National Institutes of Health, USA) (Fig. 1C). We then concatenated the separate regions of the TM together to create a rectangular image showing the tracer distribution around the full circumference of the TM (Fig. 1D). The same process was applied to both tracer images simultaneously, and we generated separate images for each tracer colour (Fig. 1E). All pixels lying outside the TM domain, as defined by the selected polygons described above, were assigned as not-a-numbers (NaNs) in MATLAB and were excluded from the calculations of average tracer intensity as described below.

To account for differences in brightness between the two tracer patterns, the blue tracer was multiplied by the following scaling factor that minimises the mean squared error between the two tracers patterns (Chang et al., 2014):

$$\mathrm{scaling factor}=\frac{\sum R_{i}B_{i}}{\sum B_{i}^{2}}$$

where *R_i_* and *B_i_* represent the red and blue tracer intensity of the *i^th^* pixel, respectively, summed over all pixels within the domain of the TM. This scaling factor increases the average intensity of the blue tracer while preserving the spatial variations in tracer intensity to give the best possible correlation in a least-squared sense between the two tracer patterns.

**Fig. 2.**
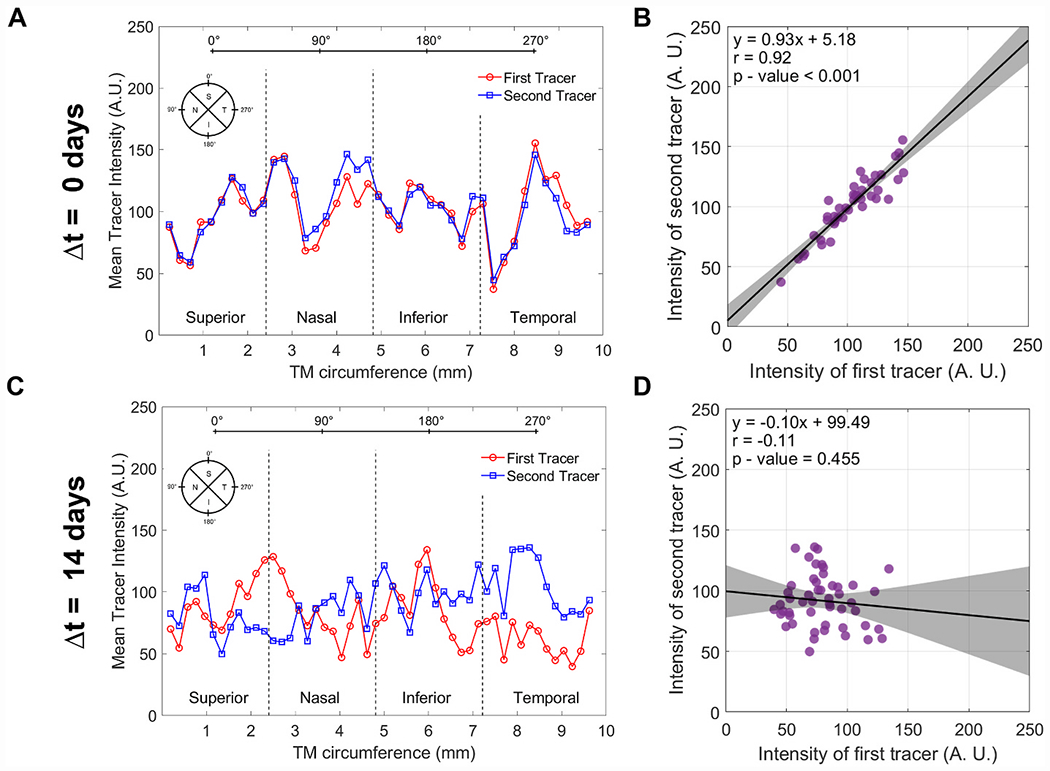
Analysis of the tracer intensity profile around the TM circumference and the spatial correlation between the two tracer profiles. For each eye, we assessed the spatial variations in the tracer intensity profile along the outflow pathway (A, C) and the co-localisation of both tracers based on linear regression of the average tracer intensities within each bin (B, D). Panels A and B show a representative example where tracers were delivered simultaneously and patterns colocalise and are highly correlated. In contrast, panels C and D show an example where tracers were delivered two weeks apart, where patterns do not colocalise and exhibit no significant correlation. Each data point represents the average tracer intensity for an individual bin. In panels B and D, the black lines and shaded regions indicate the best fit linear regressions (given by equation and associated parameters) and 95% confidence intervals, respectively. The tracer intensity profiles shown here correspond to the tracer images for Δt = 0 days and Δt = 14 days shown in Fig. 3.

The TM band was then divided into rectangular bins for analysis (45 ± 6 bins/eye; mean ± SD; n = 39 eyes), which allowed us to analyse variations in tracer labelling patterns over tissue-scale dimensions (as opposed to variations over individual pixels) that are likely to be more relevant for segmental outflow. The bin width was 212.0 ± 28.2 μm (163 ± 22 px) and the bin height was 277.8 ± 49.1 μm (214 ± 38 px), spanning the full anterior-posterior dimension of the TM (Fig. 1F). The same binning procedure was applied to both tracer images. For each bin, we calculated the average fluorescence intensity for each tracer, including data from only those pixels within the TM domain and excluding all NaNs outside the TM domain (see above). Plotting the average intensity of each tracer per bin as a function of distance yields the “tracer intensity profile” that reflects variations in segmental outflow around full circumference of the TM (Fig. 2 A, C). We defined the location of the TM corresponding to the superior-most or inferiormost position based on the location of the needle injection site (see above), and used this location to demarcate the nasal, temporal, inferior and superior quadrants.

To quantify how the tracer labelling patterns changed over time, we performed spatial co-localisation analysis and calculated the Pearson’s correlation coefficient, *r*, between the two tracer intensity profiles for each eye analysed by bin. Specifically, we plotted the average intensity of one tracer versus the intensity of the other for each bin and applied linear regression analysis to calculate *r*, which represents the strength of the spatial correlation. Briefly, *r* = 1 represents perfect correlation, meaning that the two tracer patterns are spatially identical, and *r* = 0 representing no correlation, or zero relationship, between the two tracer patterns (Fig. 2 B, D). A value of *r* = −1 means that the two tracer patterns are anti-correlated, when one tracer pattern is the exact opposite of the other. In summary, a value of *r* near one indicates that the two tracers tend to accumulate in the same regions of the TM, with little difference between the two tracer labelling patterns (as in Fig. 2 A, B). In contrast, a value of *r* near zero indicates that the two tracers tend to accumulate in different regions of the TM, with a significant difference between the two tracer labelling patterns (as in Fig. 2 C, D).

Statistical significance was analysed using an unpaired two-sample two-tailed Student’s t-test when comparing between two groups. For determining whether measured values of *r* were significantly different from the value of zero, we used a one-sample two-tailed Student’s t-test. The significance threshold was set to 0.05.

## 3. Results

### 3.1. Segmental outflow patterns in young adult mice

**Fig. 3.**
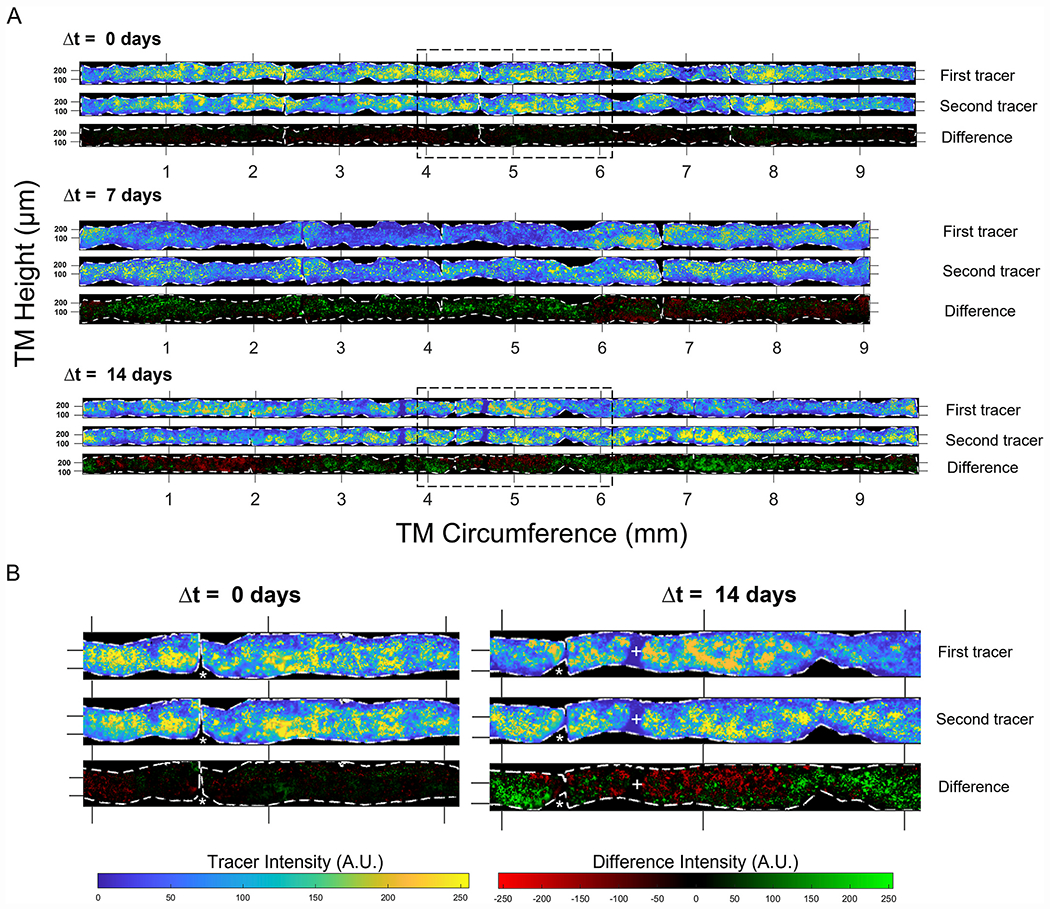
Tracer labelling patterns in the TM change over time in living mice. (A) Representative images of tracer labelling patterns in the TM from three different eyes of 3-month-old mice, where tracers were delivered at Δt = 0, 7, or 14 days apart. The signal from each tracer was straightened (see Fig. 1) and the fluorescent intensity was pseudocoloured to display low pixel values in blue and high pixel values in yellow, independent of the actual tracer colour. Black pixels lie outside of the TM domain, which is demarcated by the dashed white borders. The difference images show the result of subtracting the first tracer image from the second, with green (or red) regions representing an increase (or decrease) in the second tracer relative to the first. The images shown for Δt = 0 days and Δt = 14 days coincide with the tracer intensity profiles and correlations shown in Fig. 2. (B) Magnified images from boxed regions of panel A to show the fine detail of the tracer labelling patterns in the TM. Asterisks represent gaps in the TM domain where an incision was made between quadrants in the flat-mount image (see Fig. 1). Regions of consistent low tracer intensity are indicated by + symbols. Ticks along the horizontal or circumferential axis demarcate steps of 1 mm, while the ticks on the vertical or anterior-posterior axis demarcate steps of 0.1 mm.

We examined tracer labelling patterns in straightened images of the TM, where the vertical and horizontal axes of the straightened images represented the anterior-posterior height and circumferential directions, respectively, relative to the TM (Fig. 3). In the straightened images, one can visually appreciate that the tracer patterns exhibit clusters of high, intermediate, and low tracer labelling, consistent with the notion of segmental outflow. There was no consistent trend for higher or lower tracer labelling in any one quadrant, nor was there a consistent labelling pattern across quadrants that was preserved between paired eyes (Supplemental Data). We next examined how these segmental outflow patterns changed over time within individual mice.

When two tracer colours were delivered simultaneously (Δt = 0 days), there was little discernible difference between the two tracer patterns (Fig. 3A, top). Regions of the TM that were intensely labelled by the first tracer tended to be intensely labelled by the second. Focussing on the fine granularity in the tracer labelling, it was possible to identify similar granulation patterns in both tracer images. Subtracting the second tracer image from the first yielded a difference image that was largely featureless for Δt = 0 days, as the two tracer labelling patterns were highly similar (Fig. 3B).

At Δt = 14 days, however, the tracer labelling patterns were noticeably different between the two time points (Fig. 3A bottom). Large regions that were intensely labelled by one tracer often appeared weakly labelled by the other tracer. Despite such differences in local tracer intensity, it was possible to identify granulation patterns that were spatially preserved in both tracer images, suggestive of common outflow routes that exhibit different levels of filtration between the two time points. Taking the difference between the two tracer patterns revealed large swaths of the TM where tracer labelling either increased (indicated by green pixels in the difference images in Fig. 3) or decreased (indicated by red pixels) between the two time points (Fig. 3B).

**Fig. 4.**
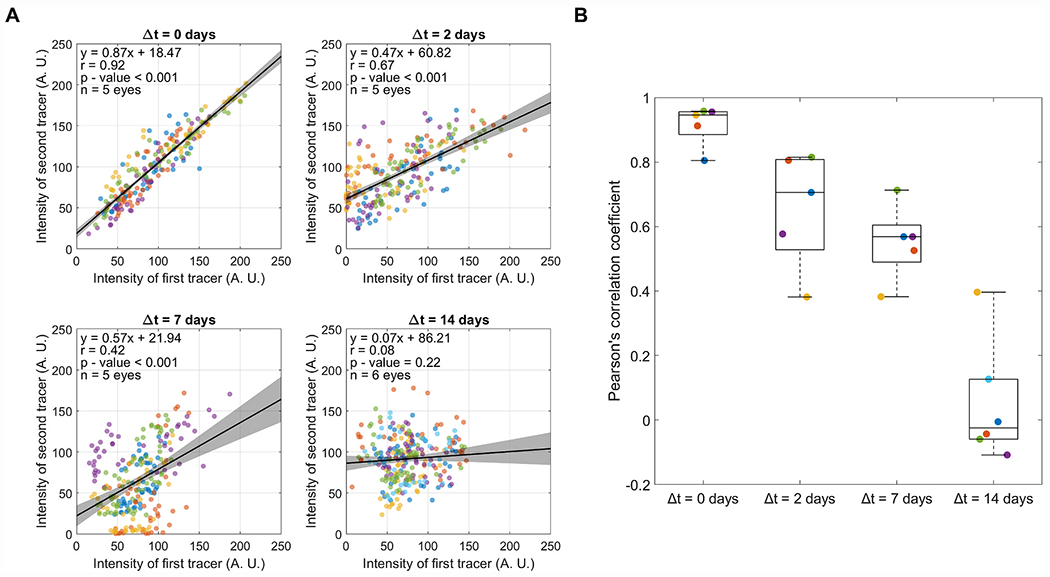
The decorrelation of the two tracer-labelling patterns over time in living mice. For tracers delivered at different time intervals (Δt = 0, 2, 7 and 14 days), we calculated the average intensity for each tracer in each bin, and we plotted the average intensity of the second tracer relative to that of the first. (A) Linear regression between the two tracer patterns, with each data point representing the averages from an individual bin and marker colours indicating separate eyes. All data from all eyes were pooled to calculate the Pearson’s correlation coefficient (*r*) displayed on each plot. Black lines and shaded regions indicate the best fit linear regression (given by equation) and 95% confidence intervals, respectively. (B) The decay in the Pearson’s correlation coefficient over time. Data points show *r* for individual eyes, and the box-and-whisker plots represent the interquartile range (box), median (centreline) and full range of data (whiskers). Marker colours are preserved between panels A and B for each value of Δt, such that data points of the same colour are obtained from the same eye in panels A and B for a given value of Δt.

At Δt = 0 days, there was a tight correlation between the two tracer patterns, as expected for simultaneous delivery, with *r* = 0.95 [0.84, 0.99] (mean [95% confidence interval], n = 5 eyes; Fig. 4). At Δt = 2 days, *r* had decreased to 0.66 [0.43, 0.88] (n = 5 eyes), and at Δt = 7 days, *r* was 0.55 [0.40, 0.70] (n = 5 eyes). Up until at least Δt = 7 days, the value of *r* was statistically different from zero (p < 0.001), and we observed a statistically significant decrease in *r* with increasing Δt (p < 0.001). However, at Δt = 14 days, *r* was no longer different from zero (*r* = 0.05 [−0.14, 0.25], n = 6 eyes, p = 0.53), indicating a complete loss of spatial correlation between the two tracer patterns (Fig. 4). This time-dependent decrease in spatial correlation was preserved even if the bin size was expanded to encompass entire quadrants (Supplemental Data).

**Fig. 5.**
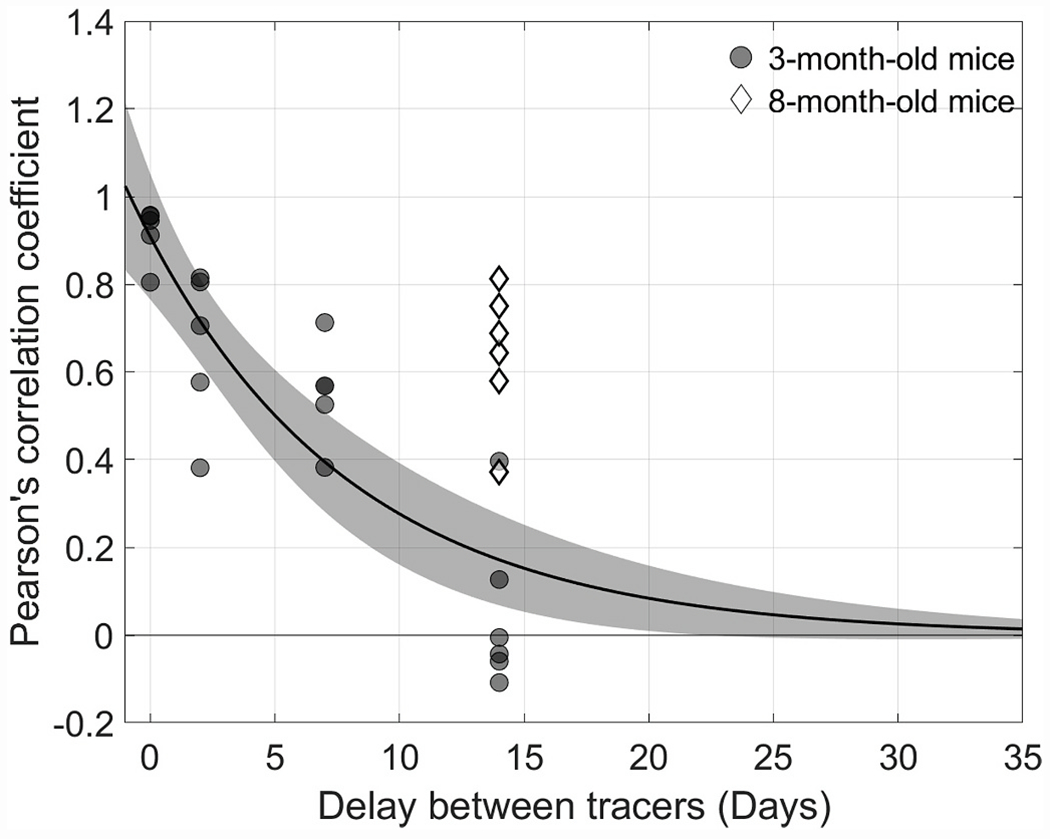
Decay of the Pearson’s correlation coefficient as a function of the time interval, Δt, between the injection of the two tracers for 3-month-old mice (circles). The black curve and shaded regions indicate the best exponential fit and 95% confidence intervals, respectively. The two tracer patterns decorrelate with a half-life of about 5 days for young adult mice. For 8-month-old mice, however, after Δt = 14 days (diamonds) the Pearson’s correlation coefficient lies well outside of the confidence intervals delineating the decay observed in 3-month-old mice, suggesting that segmental outflow patterns change more slowly in the TM of older mice. The *r* value in older mice at Δt = 14 days is nearly equivalent to that measured at approximately Δt = 2 days in younger mice.

We then fit the decrease in *r* versus Δt to an exponential decay function. This yielded an estimate of the half-life of the correlation coefficient at 5.0 [3.5, 7.4] days (n = 39 eyes, Fig. 5). This time-dependent decrease in *r* could not be attributed to a loss, or flushing out, of tracer from the TM, as there was no detectible change in the average tracer intensity (or its variance) as a function of time that tracer was retained within the TM (Supplemental Fig. 3). Further, the tight correlation between tracers delivered simultaneously (Δt = 0 days) was preserved even if the tracer was retained *in vivo* for 14 days, suggesting that the time-dependent decay in *r* was not attributable to a random spatial redistribution of tracer-labelling patterns over time once tracer is deposited within the TM (Supplemental Fig. 4). These data reveal that segmental outflow patterns are not static in living mice but rather change over time as regions of active filtration become redistributed spatially within the TM over a period of several days.

### 3.2. Segmental outflow patterns in older mice

**Fig. 6.**
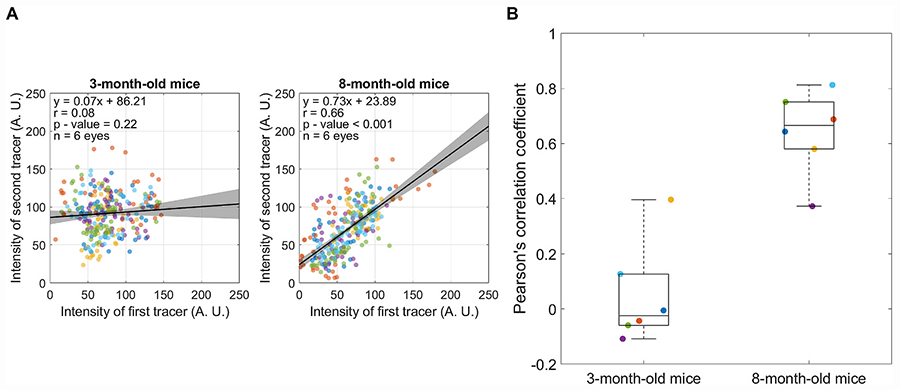
Comparison of the Pearson’s correlation coefficient (*r*) between 3-month-old and 8-month-old mice when tracers were delivered with a time interval of Δt = 14 days. (A) Linear regression between the two tracer patterns, with each data point representing the average tracer intensities from individual bins and marker colours indicating separate eyes. All data were pooled to calculate the value of *r* displayed on each plot. Black lines and shaded regions indicate the best fit linear regression (given by equation) and 95% confidence intervals, respectively. (B) There is a significant difference between the value of *r* measured in older mice versus that measured in younger mice (p < 0.001). Data points represent *r* for individual eyes, and the box-and-whisker plots represent the interquartile range (box), median (centreline) and full range of data (whiskers). Marker colours are preserved, such that data points of the same colour are obtained from the same eyes in panels A and B.

We then repeated the tracer experiment in six eyes from three live mice aged 8 months, fixing the interval between tracer infusions at Δt = 14 days. We did not observe obvious differences in the quality of the tracer labelling patterns in the TM of older mice relative to that of younger mice. High, intermediate, and low regions of tracer labelling were still observed (Supplemental Fig. 5). However, in contrast to the findings in young mice, the two tracer patterns remained significantly correlated in older mice (*r* = 0.64 [0.48, 0.80], n = 6 eyes; p < 0.001). This value of *r* measured in older mice was significantly larger than that measured in young mice at Δt = 14 days (p < 0.001) and was indistinguishable from that measured in younger mice at Δt = 2–7 days (p = 0.88 and p = 0.32 respectively; Figs. 5 and 6). These data reveal that the rate at which segmental outflow patterns become redistributed in the TM appears to slow with age.

### 3.3. In vivo segmental outflow patterns are largely preserved ex vivo

**Fig. 7.**
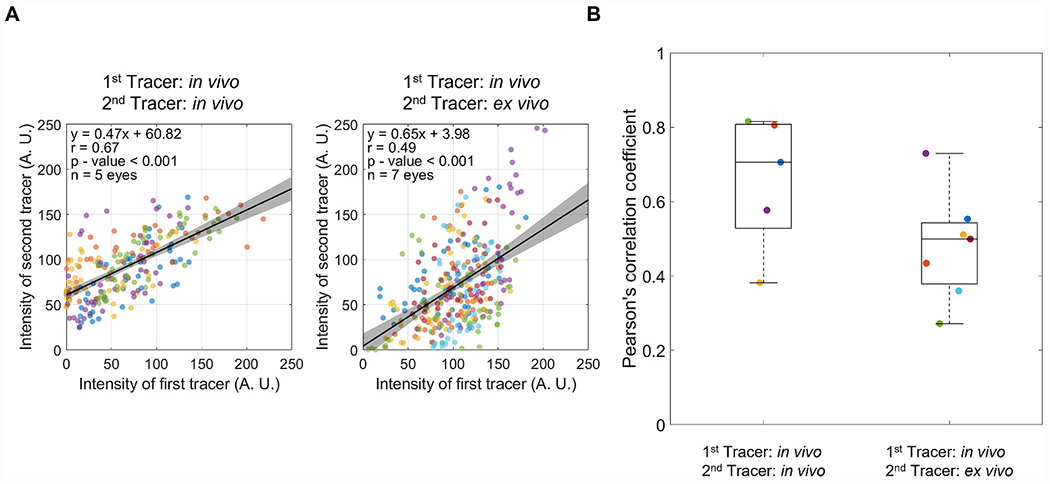
Comparison of the Pearson’s correlation coefficient (*r*) between eyes that had both tracers delivered *in vivo* with a time interval of Δt = 2 days versus eyes that had the first tracer delivered *in vivo* and the second tracer delivered *ex vivo* 2 days later. (A) Linear regression between the two tracer patterns, with each data point representing the average tracer intensities from individual bins and marker colours indicating separate eyes. All data were pooled to calculate the value of *r* displayed on each plot. Black lines and shaded regions indicate the best fit linear regression (given by equation) and 95% confidence intervals, respectively. (B) There is no significant difference between the value of *r* measured when comparing *ex vivo* versus *in vivo* with Δt = 2 days against *r* measured entirely *in vivo* with Δt = 2 days (p = 0.09). Data points represent *r* for individual eyes, and the box-and-whisker plots represent the interquartile range (box), median (centreline) and full range of data (whiskers). Marker colours are preserved, such that data points of the same colour are obtained from the same eyes in panels A and B.

Most prior reports of segmental outflow were based on *ex vivo* tracer studies. An important unanswered question is whether segmental outflow patterns mapped *ex vivo* reflect those occurring *in vivo*. To address this question, we infused one tracer colour into both eyes of four living mice under anaesthesia. Two days later, the mice were humanely culled, and both eyes were enucleated for *ex vivo* infusion with a second tracer colour. Comparing the spatial correlation between the two tracer patterns yielded a Pearson’s correlation coefficient that was significantly larger than zero (*r* = 0.48 [0.34, 0.62], n = 7 eyes; p < 0.001, Fig. 7). Further, the correlation coefficient was not significantly different from that measured entirely *in vivo* with the same interval of Δt = 2 days (*r* = 0.66 [0.43, 0.88]; p = 0.09). These data suggest that segmental outflow patterns measured *ex vivo* largely reflect those that are present *in vivo* at the time of death.

## 4. Discussion

### 4.1. Segmental outflow is dynamic in mice, but these dynamics slow with age

Aqueous humour does not drain uniformly through the TM, but rather follows preferential routes, such that only a portion of the TM – approximately one-third in humans (Chang et al., 2014; Vranka et al., 2015) – is filtration-active at any given instant. In this study, we infused fluorescent tracer microparticles into the anterior chamber of living mice to label filtration-active or “segmental” routes of aqueous humour drainage through the TM. By comparing segmental outflow patterns labelled at different time points, spanning 2 days to 2 weeks, we were able to show that the filtration-active regions of the TM are not fixed or static, but redistribute over time, such that regions of the TM that are filtration-active at one instant are not necessarily filtration-active at a later instant.

In healthy 3-month-old mice, the spatial redistribution of segmental outflow patterns occurs over a time scale of several days, such that by 14 days, segmental outflow patterns have completely decorrelated from those labelled 2 weeks earlier. In 8-month-old mice, segmental patterns were more consistent over time, such that after an interval of 14 days, the change in tracer patterns measured in the older cohort was comparable to that measured after 2–7 days in the younger cohort. Thus, there appears to be an age-dependent decline in the rate at which segmental outflow patterns are redistributed spatially within the TM of living mice.

In contrast to the findings reported in this study, Vranka and colleagues reported negligible changes in segmental outflow patterns within the TM of human eyes over a 7 day period of organ culture perfusion at a physiological pressure drop of 8.8 mmHg (Vranka et al., 2020). When the pressure drop was doubled to 17.6 mmHg, significant changes were observed in the segmental outflow routes over the same time interval, suggesting that the tissue retained the ability to respond to pressure stimuli. Perhaps this suggests that living mice exhibit more dynamic segmental outflow relative to enucleated human eyes. Alternatively, the older human eyes used by Vranka et al. (2020) (65–90 years, with an average of 77.6 years), could have already experienced an age-related decline in how segmental outflow patterns change over time, similar to the findings observed in the older mice in the current study. Future studies should examine whether segmental outflow patterns change over time in human anterior segments isolated from younger donors.

### 4.2. Use and assumptions of fluorescent tracer microparticles for labelling segmental outflow

Tracer-labelling patterns are presumed to reflect the segmental or non-uniform patterns of aqueous humour filtration through the TM, with regions that are more intensely labelled with tracer coinciding with regions of greater local filtration velocity. This interpretation is supported by studies in human eyes showing that static incubation in medium containing cationic ferritin yields more uniform tracer labelling patterns than those observed following perfusion (Hann et al., 2005). This implies that preferential or heterogeneous binding patterns cannot explain the non-uniform labelling of tracer in the TM. Consistent with this notion, recent studies have reported that the labelling pattern of fluorescent tracer microparticles in the TM matches the patterns visualized by aqueous angiography (Saraswathy et al., 2020).

As the tracer is carried into the TM by flow, individual microparticles are left behind to decorate the surface of cells or to become entrapped within extracellular matrix. As most cell surfaces and matrix proteins are negatively charged, we (Braakman et al., 2015; Chang et al., 2014) and others (Cha et al., 2016; Swaminathan et al., 2013) have opted for negatively charged (at physiological pH) carboxyl- or sulphate-modified tracer microparticles so as to minimise charge-charge interactions that may adversely affect outflow function. For example, positively charged ferritin tracer has been shown to reduce outflow facility, while negatively charged ferritin does not (Ethier and Chan, 2001).

By perfusing different colours of fluorescent tracer microparticles at different time points, one may examine how segmental outflow patterns change in response to an experimental stimulus (Chang et al., 2014; Zhu et al., 2010, 2013). Importantly, the first tracer pattern serves as an internal control or reference for the second tracer pattern, allowing sensitive detection of changes in local filtration patterns in response to a stimulus. In the current study, we extended this two-colour technique to examine time-dependent changes over a period of days to weeks, and we applied the technique to living mice. Although segmental outflow has been reported before in living mice, prior studies examined tracer labelling patterns only at a single time point (Carreon et al., 2017; Li et al., 2016; Swaminathan et al., 2013).

The key assumptions underlying the two-colour tracer approach applied over intervals of time are (*i*) that the pattern of tracer labelling, once deposited in the TM, remains stationary; (*ii*) that the intensity of tracer labelling either does not decay over time (or decays uniformly throughout the TM); (*iii*) that the labelling pattern of the second tracer is not affected by the labelling pattern of the first tracer; and (*iv*) that the tracer labelling pattern is independent of the mode of tracer infusion or by sedimentation, which is an assumption that also applies for single time-point labelling. A corollary to assumption (*iii*) is that the supply of first tracer is absent entirely from the anterior chamber by the time the second tracer is administered, which can be accomplished *ex vivo* by anterior chamber exchange or *in vivo* by allowing sufficient time for aqueous humour turn-over in the anterior chamber.

We observed no detectible decrease in the average or spatial variance of tracer intensity as a function of time that tracer microparticles were retained in the TM of living mice. Thus, there does not appear to be any significant loss, clearance or flushing away of tracer microparticles due to natural outflow of aqueous humour *in vivo* over the period of 14 days. Further, the relative consistency in tracer labelling patterns over an interval of 2 days suggests that deposition of the first tracer does not significantly influence the labelling patterns of the second.

We administered the tracers under constant pressure intracameral infusion for a fixed duration, as opposed to constant volume injection, to avoid pressure spikes that could potentially affect segmental outflow. Our approach has the potential to introduce variations in total amount of tracer infused into each eye and deposited into the TM, but we do not expect variations in the quantity of tracer to have a significant effect on the spatial patterns of tracer labelling. This is because, regardless of differences in the overall average tracer intensity, our analysis focusses on spatial variations in local tracer intensity, which should be independent of differences in overall quantity of tracer delivered to the TM. We also compensate for differences in the average intensity between the two tracer colours by applying a multiplicative scaling factor applied to the blue tracer that minimises the mean squared difference between both tracer patterns (i.e., yielding the best possible correlation between the two tracer patterns).

### 4.3. Segmental outflow in vivo versus ex vivo

Previous studies have described non-uniform or segmental outflow in the TM of multiple species, including monkey (Epstein and Rohen, 1991; Sabanay et al., 2000), human (Braakman et al., 2015; Cha et al., 2016; Chang et al., 2014; de Kater et al., 1989; Ethier and Chan, 2001; Hann et al., 2005; Hann and Fautsch, 2009; Keller et al., 2011; Swain et al., 2022; Yang et al., 2013), mouse (Carreon et al., 2017; Li et al., 2016; Swaminathan et al., 2013) and bovine (Battista et al., 2008; Lu et al., 2008; Zhu et al., 2010). However, with few exceptions (Carreon et al., 2017; Li et al., 2016; Swaminathan et al., 2013), most prior studies have examined tracer labelling patterns in enucleated eyes. An important question, therefore, is whether segmental outflow truly occurs *in vivo* or whether the appearance of segmentation could be attributable to enucleation or post-mortem changes in the eye or outflow pathway. Such changes may include reduction of ciliary muscle tone, loss of distal vascular tone, or elimination of the downstream pressure boundary conditions in the distal/episcleral vessels. There may also be post-mortem changes in the TM or Schlemm’s canal, but these should be minimal for eyes that are freshly enucleated and perfused soon after death, particularly if performed within a time window when metabolic-dependent outflow is retained, as has been shown in studies with similar post-mortem times (Reina-Torres et al., 2020).

In this study, we used the two-colour technique to directly compare segmental outflow patterns labelled *in vivo* with patterns labelled *ex vivo* in the same eye after an interval of 2 days. Although the patterns were not identical, the correlation was significantly greater than zero and not detectibly different from the correlation measured when two tracers were infused *in vivo* with the same 2-day interval. This suggests that segmental outflow patterns measured *ex vivo* are largely consistent with those present *in vivo* at the time of death, although a more focused study with a shorter interval between the *in vivo* and *ex vivo* tracer infusions would be warranted. Regardless, these results confirm that segmental outflow is not an artefact of enucleation, consistent with prior reports of segmental outflow in living mice (Carreon et al., 2017; Li et al., 2016; Swaminathan et al., 2013). Moreover, these results demonstrate that post-mortem changes, such as loss of ciliary muscle tone or alterations in distal vasculature (see below) appear to have relatively little effect on segmental outflow patterns.

### 4.4. Role of distal vessels in segmental outflow

In this study, we restricted our attention to segmental outflow patterns in the TM, although we recognise that other groups have reported non-uniform outflow in the distal intrascleral or episcleral vessels. After perfusion with fluorescent tracer microparticles Cha et al. (2016), examined the distal outflow vasculature of enucleated human eyes, reporting a non-uniform distribution of tracer labelling with regions of higher tracer labelling in the episclera coinciding spatially with regions of higher tracer-labelling in the TM. Using similar techniques, Ren et al. (2016) reported a broadening of tracer labelled regions in distal vasculature and a dilation of episcleral vessels following treatment with netarsudil. Using aqueous angiography, Huang and colleagues visualized the non-uniform distribution of molecular tracers, specifically fluorescein or indocyanine green, in the episclera of human patients (Huang et al., 2017a) and living monkeys (Huang et al., 2017b), as well as in enucleated pig and human eyes (Huang et al., 2016; Saraswathy et al., 2016, 2020). In living eyes, aqueous angiography revealed the highly dynamic and pulsatile appearance of tracer labelling within the episclera that varied over time scales of seconds to minutes (Huang et al., 2017a, 2017b).

An important question is whether or how the distal outflow vasculature affects segmental outflow patterns and their dynamics in the TM. For instance, previous studies have reported that regions of higher tracer labelling in the TM tend to coincide with regions nearer to collector channel ostia (Battista et al., 2008; Chang et al., 2014; Hann and Fautsch, 2009; Zhang et al., 2009). As the distal vasculature likely represents a significant fraction of total outflow resistance (Rosenquist et al., 1989), it is reasonable that the anatomy of the distal vascular network would have some imprint on segmental outflow patterns upstream in the TM. Along these same lines, time-dependent changes in segmental outflow may be attributed to alterations in distal vessel calibre, vasodilation, or vasoconstriction, which may be actuated by perivascular smooth muscle cells (de Kater et al., 1990; Overby et al., 2014) and their associated innervations (Selbach et al., 2005). Alternatively, intrascleral arterio-venous anastomoses, such as those described in non-human primates (Funk and Rohen, 1996; Selbach et al., 2005), may modulate the pressure distribution within the distal vascular network to affect changes in segmental outflow. As these mechanisms may act within seconds, it seems more likely that rapid modulation of distal vessel tone could potentially explain the rapid second-to-minute scale changes in tracer distribution reported within the episclera of living primates (Huang et al., 2017a, 2017b). In contrast, it seems unlikely that relatively fast changes in distal vessel tone could explain the time-dependent changes in segmental outflow observed over several days in living mice, but perhaps there is some additional physiological mechanism that modulates distal vessel resistivity over day-long time scales.

We did not attempt to correlate segmental outflow patterns in the TM with distal vessel anatomy. However, following death and enucleation, the eyes used for *ex vivo* tracer infusion would have likely experienced profound changes in local resistivity and pressure distribution within the distal vascular network, despite enucleated eyes maintaining some degree of distal vasoactivity in response to soluble factors (McDonnell et al., 2018). Thus, if the distal vasculature had a significant role in determining segmental outflow patterns in the TM, we would suspect that the process of enucleation, by disrupting the distribution of pressure and resistivity within the distal vasculature, would have significantly altered the tracer labelling patterns in the TM between *in vivo* and *ex vivo*. However, the relative consistency in tracer labelling patterns observed *in vivo* versus *ex vivo* with a time interval of 2 days, and their similarity with pattern changes measured entirely *in vivo* with the same time interval, suggests that the distal vasculature has a relatively small contribution towards the time-dependent change in segmental outflow patterns change within the TM, at least in mice. Further work is needed to clarify the role of the distal vasculature on segmental outflow.

### 4.5. The factors controlling segmental outflow dynamics in the TM

The factors controlling segmental outflow are almost certainly important in the regulation of aqueous humour outflow resistance and IOP. It has already been proposed, for instance, that disruption of outflow segmentation in the TM may contribute to outflow dysfunction and ocular hypertension in glaucoma (de Kater et al., 1989). For these reasons, researchers have investigated the factors that correlate with tracer-labelling patterns in the TM. Vranka and colleagues investigated the relationship between local tracer labelling intensity and expression of ECM-related genes (Staverosky et al., 2020; Vranka et al., 2020), such as versican, vascular cell adhesion protein 1 and tissue inhibitor of metalloproteinase 1 (TIMP1) that appears enriched in low tracer-labelled regions (Keller et al., 2011; Saraswathy et al., 2020). Deletion of matricellular proteins that regulate ECM assembly, such as SPARC, tends to allow for a more uniform distribution of tracer labelling in the TM (Swaminathan et al., 2013). Regions of higher tracer labelling tend to coincide with regions of thicker trabecular meshwork (Cha et al., 2016) where there are fewer connections between inner wall cells and the underlying juxtacanalicular tissue and more openings below giant vacuoles (Swain et al., 2022). Regions of higher tracer labelling also coincide with regions of greater pore density along the inner wall of Schlemm’s canal (Braakman et al., 2015), whilst low tracer-labelled regions tend to exhibit elevated tissue stiffness relative to high tracer-labelled regions (Staverosky et al., 2020; Vranka et al., 2018). Thus, there appear to be many biochemical, morphological, and biomechanical alterations that correlate with local outflow segmentation in the TM.

While there are several correlates to segmental outflow in the TM, some of which are reported above, it remains unclear which (if any) of these factors are causal and which are downstream sequela that result from changes in the outflow distribution within the TM. However, based on the findings in this study, at least in young healthy mice, any causal factors (as well as any correlates) must also be dynamic to explain how segmental outflow patterns change over time within the TM. It seems almost without doubt that understanding the factors controlling segmental outflow and how these factors contribute to segmental outflow dynamics is an important area for future study.

## 5. Conclusions

In conclusion, this study demonstrates that segmental outflow patterns are dynamic and change over time in the TM of living mice. Segmental outflow patterns change over the time scale of days in young healthy mice, redistributing over a period of two weeks. This rate at which segmental outflow is redistributed within the TM appears to be reduced in older animals, suggesting an age-related decline in dynamic outflow function. We have also shown that segmental outflow patterns measured *ex vivo* are largely representative of segmental outflow patterns that existed *in vivo* near the time of death. The mechanisms that control segmental outflow dynamics are likely important components of outflow resistance generation and dysregulation of these processes could contribute to development of ocular hypertension and glaucoma.

## Supplementary Material

1

2

## Data Availability

Data will be made available on request.

## Abbreviations:

TM: trabecular meshwork
IOP: intraocular pressure
r: Pearson’s correlation coefficient
