# Supplementary material for: Segmental outflow dynamics in the trabecular meshwork of living mice
Source: Exp Eye Res. Author manuscript; Available in PMC 2023 Jun 20. (PMC10279931; doi:10.1016/j.exer.2022.109285)
Supplement: 1 [file NIHMS1908314-supplement-1.pdf]

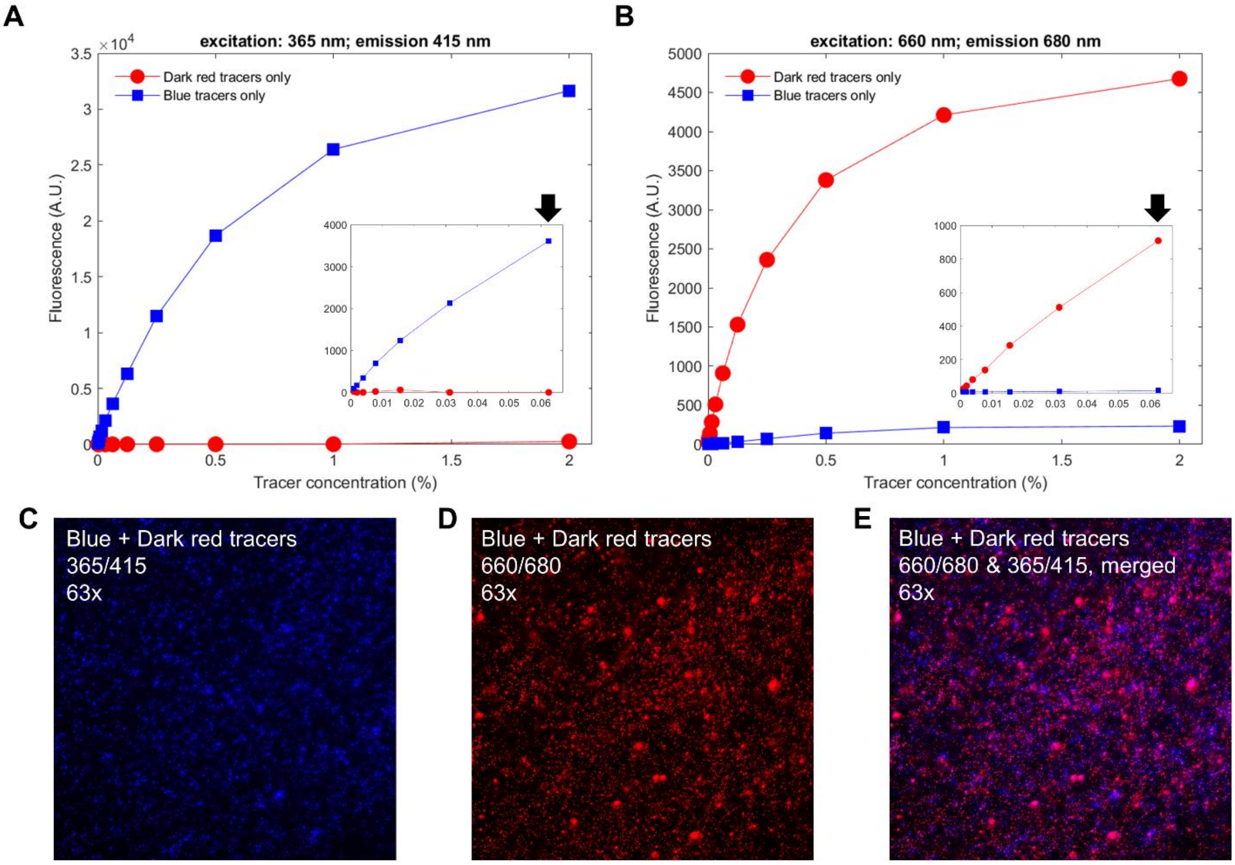

Supplemental Figure 1: The signal overlap between the two tracer colours (dark red and blue) is negligible. The fluorescent intensity of different concentrations of each tracer was measured using a Varioskan LUX plate reader (ThermoFisher Scientific, UK) set to excite and detect the emission of each tracer separately. Only signal from the blue particles was detected at 365/415 nm (A) and only signal from the dark red particles was detected at 660/680 nm (B). Insets show the lower concentrations, including the working tracer concentration infused into eyes (0.06%, arrows). Additionally, we imaged 3 µl of a mix of both tracers in suspension at 0.01% dilution each on a glass slide. The drop was allowed to dry and then covered with mounting media and a cover slip. Samples were imaged using a widefield microscope (the same microscope used to image tracers within the eyes) at 63x with immersion oil. The sample was sequentially imaged both at 365/415 nm and 660/680 nm to mimic the imaging method applied to the TM. Panel C shows the image obtain for the blue channel, panel D shows the image obtained for the red channel and panel E shows the merged image. When both tracer colours were mixed, there was no apparent colocalisation between the red and blue emissions. Based on these data, we conclude that there was no significant overlap between the two tracer colours used for this project. The emission and excitation wavelengths are also significantly removed from the emission and excitation of GFP, which may be present in the older cohort of mice.

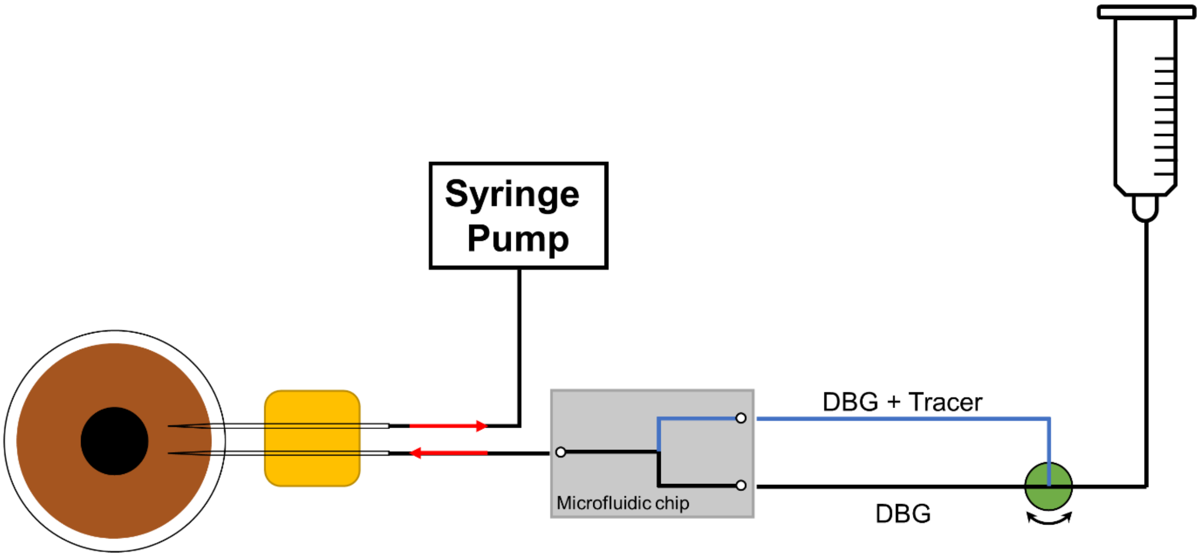

Supplemental Figure 2: Schematic representation of the perfusion set-up for *ex vivo* tracer infusion. A reservoir was connected to a microfluidic chip with two separate channels, filled with either DBG alone or DBG containing tracer. The two channels converged to flow through one of the needles used to cannulate the eye. A custom-designed holder (amber) was used to fix the distance between the two cannulas at the corneal surface and to allow for simultaneous cannulation. The green circle represents a turnable valve that directs which channel receives flow. The second needle was connected to a syringe pump set to withdrawal (at 5 µl/min for 20 minutes). This configuration allows for rapid exchange of anterior chamber contents at controlled IOP after adjusting for the hydraulic resistance of the cannula (see Methods in the main text for further details). The red arrows represent the direction of flow during the exchange. Schematic is not drawn to scale.

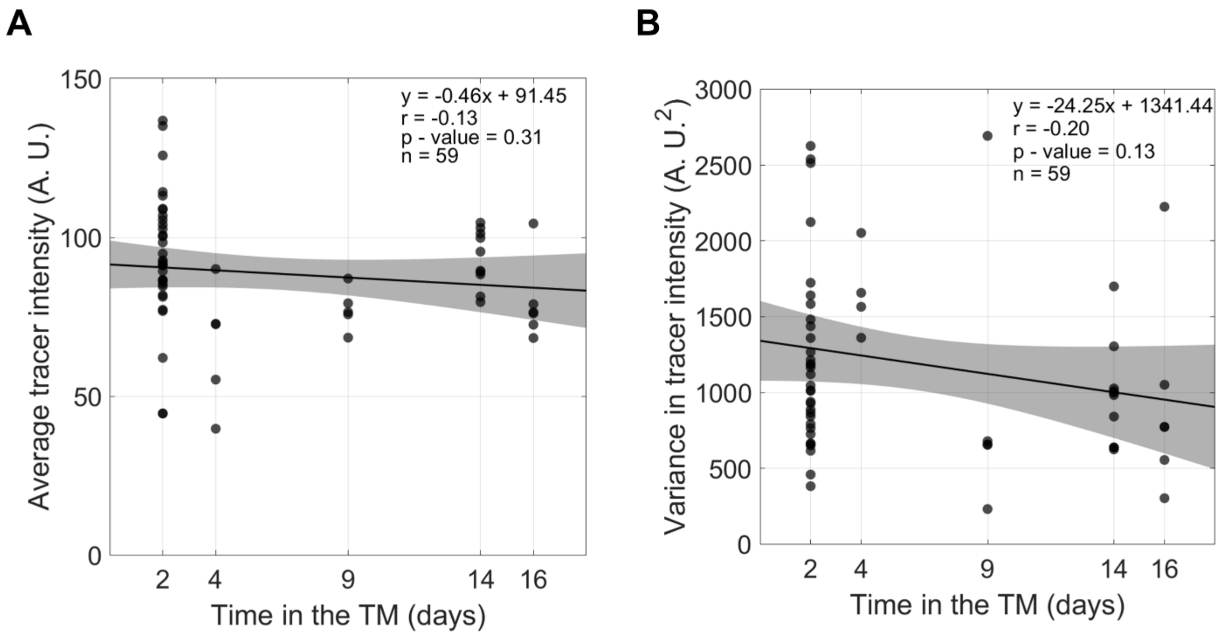

Supplemental Figure 3: To assess whether tracer microparticles were washed out of the TM over time, we examined whether there was a loss in average tracer fluorescence intensity across the entire TM (A) or a reduction in the variance of the average tracer intensity between bins (B). Neither parameter significantly declined over time (measured as the time between tracer infusion and enucleation), indicating that fluorescent tracer microparticles are not significantly lost or washed out of the TM over the time frame of these experiments. Each data point represents one tracer colour from an individual eye. Black lines and shaded regions indicate the best fit linear regressions (given by equations and associated parameters) and 95% confidence intervals, respectively.

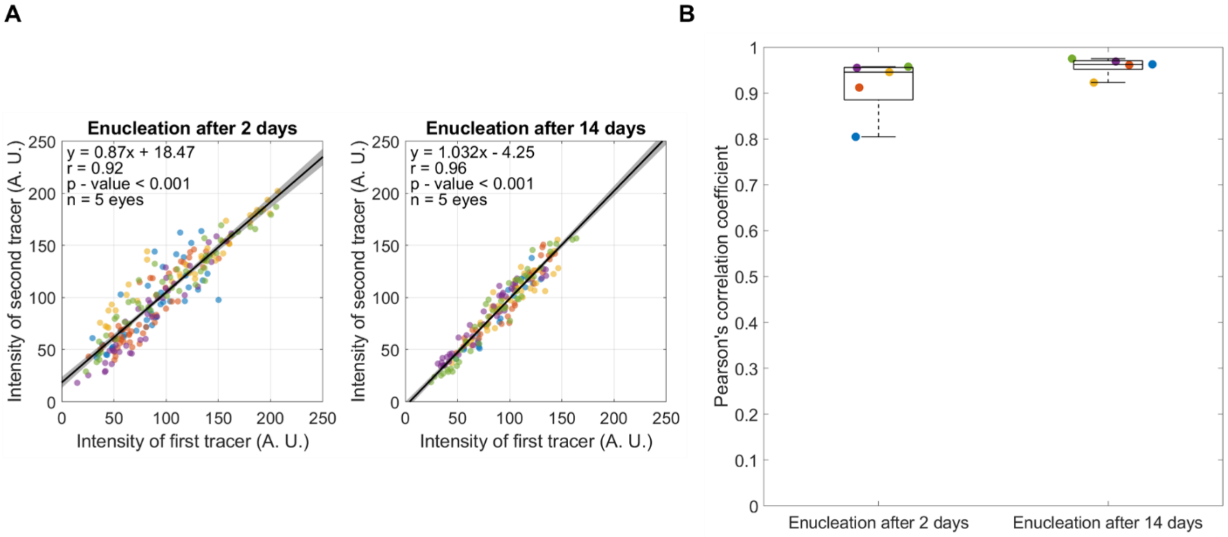

Supplemental Figure 4: To assess whether the labelling patterns of fluorescent tracer remain stationary in the TM or rather experience spatial redistribution over time after infusion, we examined the co-localisation between two tracer colours delivered simultaneously into each eye but retained *in vivo* for either 2 or 14 days. We reasoned that if significant spatial redistribution occurred within the TM (e.g., due to phagocytosis and cell migration), then we should observe a decline in the Pearson's correlation coefficient, r, as a function of time that the tracer was retained within the TM *in vivo*. This would be because any random process leading to spatial redistribution should tend to magnify over time any subtle pattern differences between two tracers that were simultaneously infused.

In each case, the signal from both tracers was highly correlated. Tracers that were retained within the TM for 2 days exhibited a correlation coefficient of r = 0.92 [0.84, 0.99] (n = 5 eyes) while tracers that were retained within the TM for 14 days had correlation coefficient of r = 0.96 [0.93, 0.98] (n = 5 eyes). There was no significant difference in the value of r between the two cases (p = 0.19).

Panel A shows the linear correlations and associated parameters when all bins from all eyes were lumped together, where each data point represents a single bin, and each marker colour represents a different eye. Black lines and shaded regions indicate the best fit linear regressions (given by equations and associated parameters) and 95% confidence intervals, respectively. Panel B shows the population statistics for the value of r for individual eyes where tracer was retained within the TM for 2 days or 2 weeks. Box-and-whisker plots represent the interquartile range (box), median (centreline) and full range of data (whiskers). Marker colours are preserved, such that data points of the same colour were obtained from the same eye in panels A and B. The data from the tracers that were retained within the TM for 2 days are reproduced from Figure 4 of the main text.

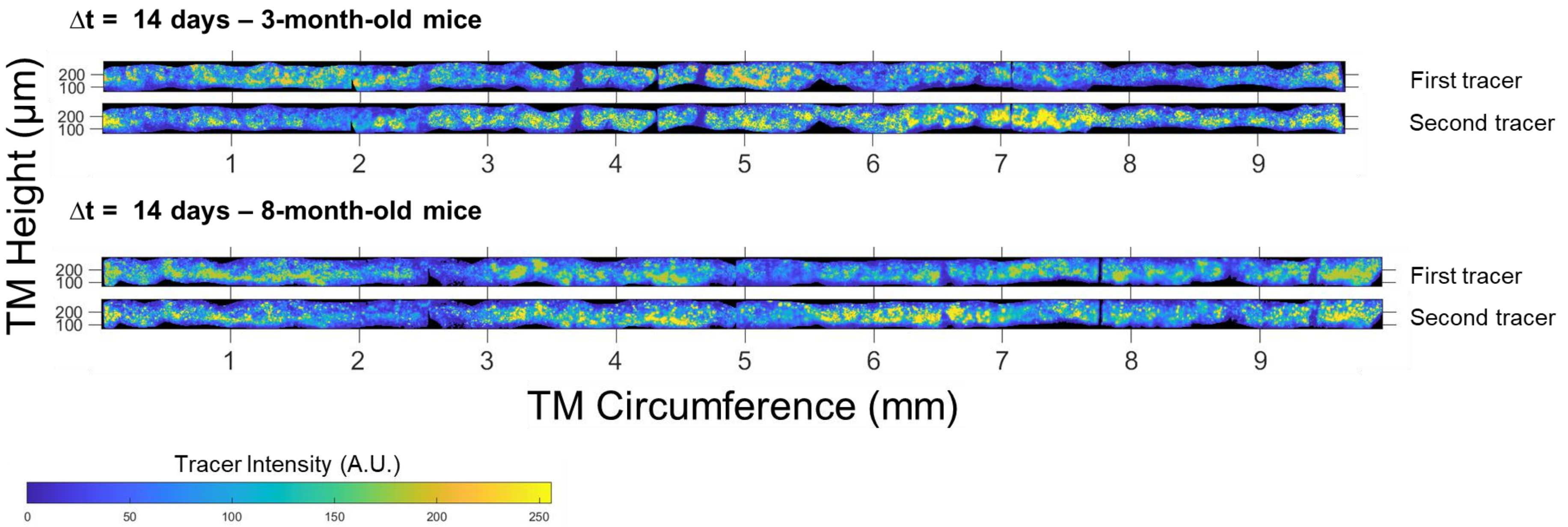

Supplemental Figure 5: Representative images of the tracer labelling patterns in the TM from two different eyes, where tracers were delivered at Δt = 14 days apart in a 3-month-old mouse (top) versus an 8-month-old mouse (bottom). The signal from each tracer was straightened and the fluorescent intensity was pseudocoloured to display low pixel values in blue and high pixel values in yellow, independent of the actual tracer colour. Black pixels lie outside of the TM domain and are excluded from analysis. Regardless of the age of the mouse, both eyes show distinctive areas of high, medium, and low regions of tracer labelling. There was no obvious qualitative difference in the tracer labelling pattern between the younger and older mice.
