# Supplementary material for: Segmental outflow dynamics in the trabecular meshwork of living mice
Source: Exp Eye Res. Author manuscript; Available in PMC 2023 Jun 20. (PMC10279931; doi:10.1016/j.exer.2022.109285)
Supplement: 2 [file NIHMS1908314-supplement-2.pdf]

Supplemental Data

Analysis of Segmental Outflow Variations by Quadrant

(Supplement to the main text titled: Segmental Outflow Dynamics in the Trabecular Meshwork of Living Mice)

Ester Reina-Torres, Tiffany MG Baptiste, and Darryl R. Overby

Imperial College London, Department of Bioengineering

Introduction

Additional analysis was performed using the fluorescent tracer intensity analysed based on quadrants rather than based on small rectangular bins (as shown in Figure 1F of main text). To perform the quadrant analysis, we identified the corneal injection sites visible in the raw fluorescent images (see Figure 1A of main text) to define the anatomical locations of the superior, inferior, temporal, and nasal quadrants (see Methods in the main text for further details). The average fluorescent tracer intensity for each quadrant was then calculated for each tracer colour. With these data we examined (i) whether there was any preferential tracer labelling by quadrant, (ii) whether tracer labelling patterns by quadrant were similar between contralateral eyes, which determines whether the paired eyes can be considered as independent samples, and (iii) whether the two tracer patterns exhibited a similar decorrelation over time (as shown in Figures 4 and 5 of main text) if the analysis were based on quadrants rather than on bins.

Is there any preferential tracer labelling between quadrants?

Other studies have reported that aqueous humour drains mainly through the nasal and inferior quadrants in human eyes (Cha et al., 2016; Hann and Fautsch, 2009), where more collector channels are found and where the trabecular meshwork (TM) appears thicker in the flow-wise direction. We wanted to assess if there is a similar preferential labelling by quadrants in mouse eyes.

Because we expect differences in quantity of tracer delivered to each eye (on account of different infusion volumes), we performed a non-parametric rank order analysis to determine if there was any preferential labelling by quadrant. For each eye, we ranked the average tracer intensity from each quadrant from the highest (rank of 1) to lowest (rank of 4) for each tracer colour. We then calculated the mean rank and performed a Kruskal-Wallis test executed in MATLAB (Supplemental Data Figure 1). Briefly, this non-parametric test examines whether the rank order samples from each quadrant are drawn from the same distribution, such that a non-significant result suggests that there is no evidence for any one quadrant having a dominant ranking. For both tracer colours, the Kruskal-Wallis test yielded insignificant results ($p = 0.6$ and $p = 0.8$ for the first and second tracers, respectively, $n = 39$ eyes each). Thus, there was no evidence of preferential tracer labelling for any given quadrant, suggesting that segmental outflow through the TM is roughly equally distributed between quadrants.

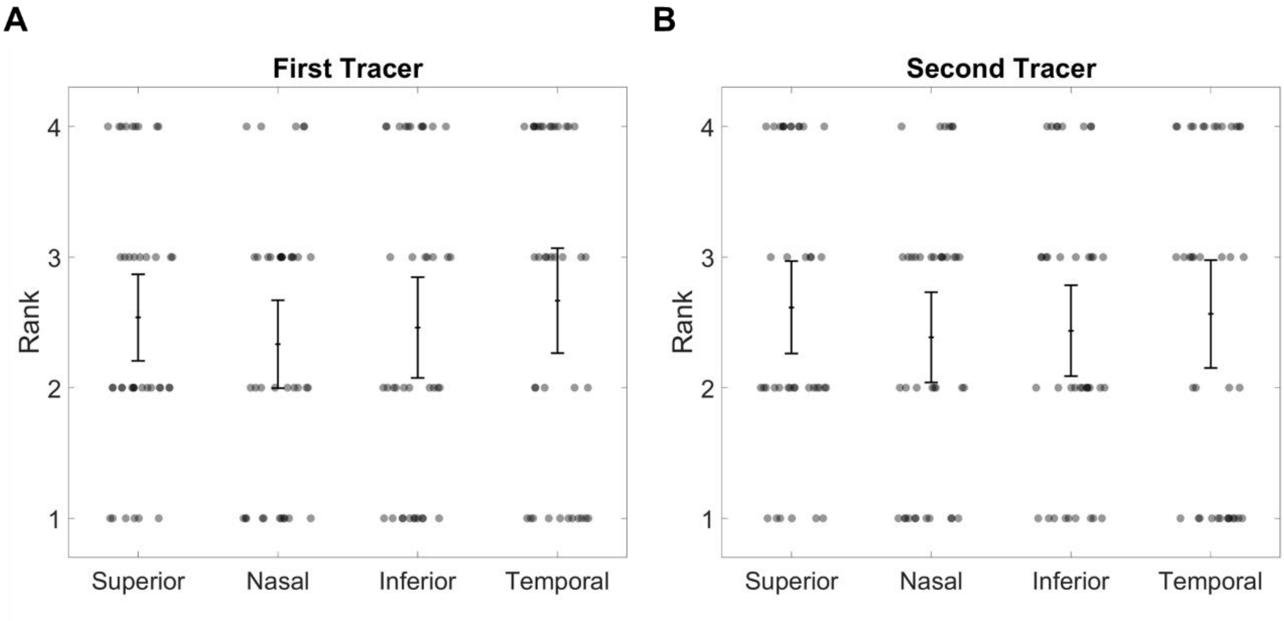

Supplemental Data Figure 1: The rank order for each quadrant for the first tracer (A) and second tracer (B). Each data point represents an individual rank order sample from an individual eye (n = 39 eyes). Error bars show the mean rank (centre tick) and 95% confidence intervals on the mean. There is no evidence for any preferential tracer labelling by quadrant.

Is there a consistent quadrant labelling pattern between contralateral eyes?

Despite there being no preferred quadrant for tracer labelling, it is still possible that a consistent preference for individual quadrants may exist between contralateral eyes. To examine this question, we used the rank order measure for average tracer intensity described above and examined the rank order of quadrants in the left eye (OS) versus that of the right eye (OD). This analysis included 9 pairs of contralateral eyes from 9 mice, comprising 72 individual quadrants. If there were a consistent trend with any one quadrant having a similar rank order in both eyes, we would expect to find a significant correlation between ranks with a slope approaching unity, representing perfect correlation. A Spearman's rank correlation analysis, however, did not detect any significant correlation between contralateral eyes, neither for the first tracer ($p = 0.52$; $n = 18$ eyes) nor the second tracer ($p = 70$, $n = 18$ eyes; Supplemental Data Figure 2). In other words, there is no evidence for any consistent trend in tracer labelling intensity by quadrant between paired eyes. Note that the time that the first tracer was retained in the TM varied between eyes, while all second tracers were retained in the TM for 48 hrs.

This analysis supports the conclusion that segmental outflow patterns are not conserved between contralateral eyes. Importantly, the lack of any detectible correlation between contralateral eyes also means that each eye of a pair can be considered as an independent sample with respect to quantification of segmental outflow patterns.

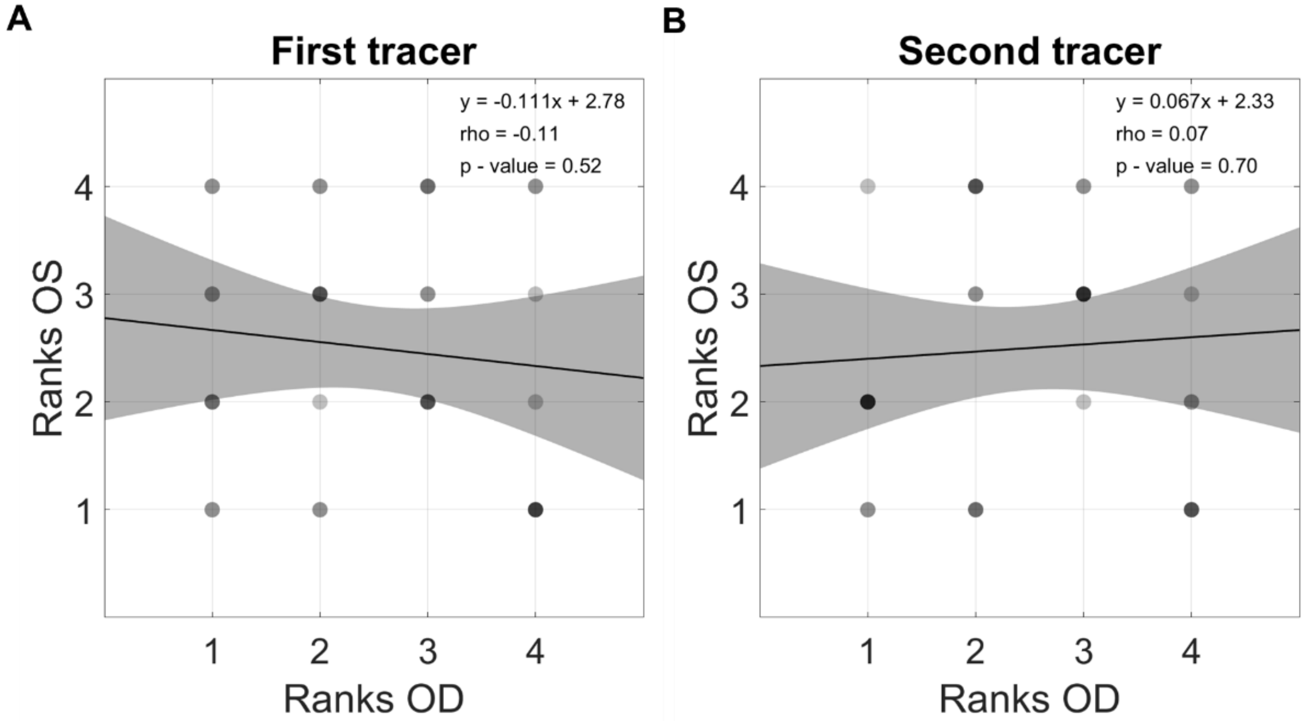

Supplemental Data Figure 2: There is no rank order correlation between contralateral eyes regarding intensity of tracer labelling by quadrant. For each eye, we measured the rank order representing the average intensity of tracer labelling for each quadrant and plotted the rank order for the left eye (OS) against that of the right eye (OD) for each quadrant. We then performed a Spearman's rank correlation, indicated by the black lines and shaded regions (indicating 95% confidence intervals). The best fit linear regression equation, p-value and rho, the Spearman's rank correlation coefficient applied to rank order data, are shown on each plot. Each data point represents one quadrant from an individual pair of eyes based on the ranking of the first tracer (A) or second tracer (B), using 18 eyes from 9 mice with 72 quadrants included for each tracer. There is no significant correlation for either case ($p > 0.5$). Darker data points represent overlapping data. For the second tracer, all data points had the same time of retention within the TM (48 hrs), while the time of retention within the TM varied between 2 days and 2 weeks for the first tracer.

Is there still a decorrelation in outflow patterns over time if the bin size were increased to encompass entire quadrants?

In the main text, we present evidence of a decorrelation between tracer labelling patterns as a function of the time interval, Δt, between tracer infusions for individual eyes (see Figures 4 and 5 of the main text). That analysis was performed using rectangular bins in the TM, as illustrated in Figure 1F of the main text. Here, we examine whether this trend depends on bin size and whether this trend is still observed when the analysis is applied to entire quadrants.

We based this analysis on the delineation of the anatomical quadrants, as defined based on the corneal injection sites (see Methods of main text) and the associated angles around the circumference of the TM (see Figure 2A,C from the main text), and we averaged the tracer intensity over each individual quadrant for each tracer colour. We then plotted, for each quadrant, the average intensity of the second tracer versus that of the first tracer and performed a linear regression analysis (as in Figure 2B,D of the main text). Similar to the results observed for rectangular bins, there was a decrease in the Pearson's correlation coefficient r as a function of Δt. At Δt = 0 days, there was a highly significant correlation between the two tracer intensities for any given quadrant, which remained statistically greater from zero until at least Δt = 7 days, although there was a time-dependent decrease in the value of r. As observed with the rectangular bins, by Δt = 14 days, there was a loss of correlation between the average tracer intensities within quadrants, as indicated by a correlation coefficient that was no longer statistically different from zero. This analysis reveals that the redistribution of segmental outflow that occurs over time in living mice does not maintain any preference for individual quadrants.

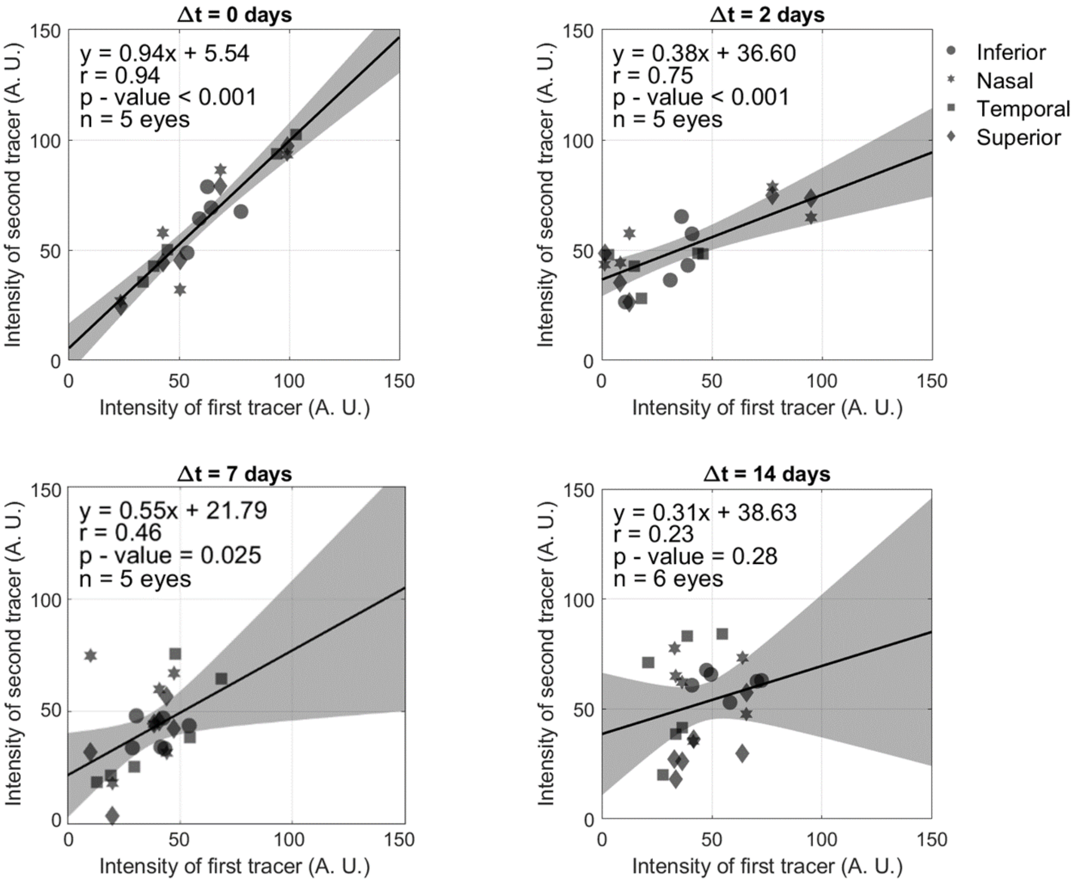

Supplemental Data Figure 3: The decorrelation of the two tracer-labelling patterns over time in living mice analysed by quadrant. For tracers delivered at different time intervals (Δt = 0, 2, 7 and 14 days), we calculated the average intensity for each tracer colour in each quadrant, and we plotted the average intensity of the second tracer relative to that of the first. We then applied linear regression analysis between the two tracer patterns, with each data point representing the averages from an individual quadrant. All data from all eyes were pooled to calculate the Pearson's correlation coefficient (r) displayed on each plot. Black lines and shaded regions indicate the best fit linear regression (given by equation) and 95% confidence intervals, respectively.

References

Cha, E.D.K., Xu, J., Gong, L., Gong, H., 2016. Variations in active outflow along the trabecular outflow pathway. Exp Eye Res 146, 354-360.

Hann, C.R., Fautsch, M.P., 2009. Preferential fluid flow in the human trabecular meshwork near collector channels. Invest Ophthalmol Vis Sci 50, 1692-1697.
